# Chronic Drug-Induced Liver Injury: Updates and Future Challenges

**DOI:** 10.3389/fphar.2021.627133

**Published:** 2021-03-08

**Authors:** Qiaoling Wang, Ang Huang, Jia-Bo Wang, Zhengsheng Zou

**Affiliations:** ^1^ Peking University 302 Clinical Medical School, Beijing, China; ^2^ Department of Liver Disease of Chinese PLA General Hospital, Fifth Medical Center of Chinese PLA General Hospital, Beijing, China; ^3^ School of Traditional Chinese Medicine, Capital Medical University, Beijing, China

**Keywords:** chronic drug-induced liver injury, definition, incidence, manifestations, glucocorticoid

## Abstract

Chronic drug-induced liver injury (DILI), defined as DILI with persistent liver injury more than one year after the first onset by the latest European guidelines, is a notable challenge globally with big issues of defining causality and establishing effective treatment. About 20% of patients with DILI develop into chronic DILI. Chronic DILI manifests as persistent or repeated inflammatory or diminishing bile ducts, even progresses to cirrhosis and needs liver transplantation eventually. However, research on chronic DILI over the last decades is still lacking, and the incidence, phenotypes, mechanisms, risk factors, and treatment have not been fully understood. In this paper, we reviewed the definition of chronic DILI, updated clinical studies in terms of incidence, special manifestations, and promising risk factors of chronic DILI, along with the recent progress and challenges in glucocorticoid therapy.

## 1 Introduction

Drug-induced liver injury (DILI) has attracted the attention of experts in the field of liver diseases and has become an important topic of discussion (Andrade and Robles-Diaz 2020; Hernandez, Pontet, and Bessone 2020; Hoppmann, Gray, and McGuire 2020). Furthermore, a variety of drugs and herbal and alternative medicines are easily accessible for patients, which may lead to a high prevalence of irregular use (Yu et al., 2017). Meanwhile, the incidence of liver damage caused by toxic substances is also on the rise (China Food and Drug Administration, 2016; Shen, Duan, and Zhuang 2015; Bjornsson 2014). Although some acute DILI cases can be severe, resulting in hospitalization and even death, the majority present as self-limited episodes, which subsides with the spontaneous normalization of liver function after stopping the insulting drugs (European Association for the Study of the Liver, 2019). In the past 30 years, the clinical practice revealed that DILI persists in some patients even though suspending the relative drugs (Lucena et al., 2011; Chalasani et al., 2014; European Association for the Study of the Liver, 2019; Yu et al., 2017). These cases may require life-long therapy to control liver inflammation, protect liver function, and maintain quality of life. The clinical manifestations of chronic DILI are diverse and complicated (Kleiner 2018). How to manage and deal with these cases is an important issue and require an approving criterion. In this paper, we reviewed the updated clinical studies in terms of definition, incidence, special manifestations, and promising risk factors of chronic DILI, along with the recent progress and challenges in glucocorticoid therapy.

## 2 Evolution of Definition

In the past 30 years, extensive exploration of DILI by global experts contributed to a better understanding of chronic DILI. The retrospective and the prospective cohort studies, as well as the real-world studies, have provided valuable information, which is useful for formulating guidelines. Looking back at the development of a definition of chronic DILI: the initial “3 months” stipulated by the Council for International Organizations of Medical Sciences (CIOMS) in 1990 (Benichou 1990) to the “12 months” stipulated by the latest European guidelines in 2019 (European Association for the Study of the Liver, 2019) (Table 1), which assists the diagnostic process of chronic DILI. The latest guidelines from the Europe group define chronic DILI as “One year after the onset of acute DILI, the biochemical indicators and/or imaging evidence of the liver continues to be abnormal", which depend on the most convincing clinical data so far (Medina-Caliz et al., 2016). In the US DILIN, any degree of elevation in any liver test lasts over 6 months can be used to suggest the development of chronic DILI (Chalasani et al., 2014). These do not seem to be in line with clinical experience because most of the abnormalities were considered clinically insignificant, and they can have a full recovery after 12 months. Usually, compared with transaminase, the normalization of enzymes that represent cholestasis is longer (European Association for the Study of the Liver, 2019) Besides, the published data of chronic DILI from China is relatively less. Meanwhile, that evidence cited in the Chinese DILI guidelines relied on follow-up data from western countries (Yu et al., 2017). Therefore, research teams in the United States and China may need a long-period follow-up to figure out the characteristics of real chronic patients and provide stricter evidence to support their views.

**TABLE1:** Evolution of definitions of chronic DILI.

Year	Source/proposer	Cutoff point of chronicity
1990	CIOMS Benichou (1990)	3M
2006	R. Andrade Andrade et al. (2006)	HC, 3 M; chol and mix, 6 M
2007	E. Björnsson Bjornsson et al. (2007)	HC, 3 M; chol and mix, 6 M
2009	DILIN Fontana et al. (2009)	6M
2011	iSAEC Aithal et al. (2011)	Persistent DILI (HC and mix, 3 M; chol, 6 M). Chronic DILI (12 M)
2014	ACG DILI Guidelines Chalasani et al. (2014)	6M
2016	R. Andrade and Spanish DILI registry Medina-Caliz et al. (2016)	12M
2017	CSH guidelines Yu et al. (2017)	6M
2018	China HILI Guidelines Wang et al. (2018)	6M
2019	EASL DILI Guidelines European Association for the Study of the Liver (2019)	12M

Definition differences of chronic DILI between European guidelines and American guidelines may directly affect the inclusion criteria of subjects in clinical studies. It is difficult to conduct region-cross prospective research. It is also not conducive to the comparison of the results. The incidence rate, predictive factors, disease mechanism, and other exploration are directly affected. Therefore, it is urgent to form an international consensus on chronic DILI.

## 3 Incidence

There is no global report on the incidence of chronic DILI. After summarizing the published data for over six-month follow-up period, we found that the incidence of chronic DILI varies greatly, ranging from 5.7% (Andrade et al., 2006) to 39% (Aithal and Day 1999) (Table 2). Recently, in a retrospective and multi-center study performed in Mainland China, 13% (Shen et al., 2019) of DILIs were chronic DILI, which is similar with the result from the US DILI Network that chronic DILI accounted for 13.6% (Chalasani et al., 2008) of all the registered DILI patients in 2008. In 2016, Medina-Caliz et al. reported that 8% (Medina-Caliz et al., 2016) of patients developed chronic DILI after one year’s follow-up. In 2014, Fontana et al. reported that the rate of chronic or persistent liver injury with six months of follow-up is 18.8% (Fontana et al., 2014) (*n* = 598) (95% CI, 15.8%–22.0%), which is close to 17.5% (Chalasani et al., 2015) that reported by Chalasani et al. in 899 consecutively enrolled patients. Then Fontana et al. continued their follow-up one year and showed the chronic incidence of 12.4% (Fontana et al., 2015). The incidence rate of 39% (Aithal and Day 1999) from the follow-up in 1999 seemed to be relatively higher than others because the data from patients who underwent liver biopsy. Similarly, Kleiner et al. also enrolled 249 patients with liver biopsies. Their result showed that more than 24% (n = 249) (Kleiner et al., 2014) of DILIs presented chronic liver histological results, including chronic hepatitis (14%) and chronic cholestasis (10%). Review of early clinical follow-up based on the criterion of “3M for HC; 6M for Mix and Chol”: Andrade et al. found that 5.7% (Andrade et al., 2006) of total idiosyncratic DILI cases (n = 493) in their 20-months follow-up registration, which is comparable to 6% (Bjornsson et al., 2007) of the chronic progress cases in another chronic clinical progress. Therefore, based on these studies, it is challenging to gauge differences in the regional incidence of chronic DILI for the difference in the definition of chronicity.

**TABLE 2:** Incidence of chronic DILI from reports.

Cut-off point of time	Incidence	Study design	Duration	Number of cases	Mean age	Time of follow-up	References
12M	39%	Retrospective	1978–1996	40	—	60M	Aithal and Day (1999)
3M for HC; 6M for mix and chol	6.0%	Retrospective	1994–2005	50	62 y	48M	Bjornsson et al. (2007)
12M	8.0%	Prospective	1994–2012	298	53 y	≥12M	Medina-Caliz et al. (2016)
3M for HC; 6m for mix and chol	5.7%	Retrospective	1995–2005	493	55.0 y	20M	Andrade et al. (2006)
6M	13.6%	Prospective	2004-2007	300	48	6M	Chalasani et al. (2008)
6M	>24.0%	Prospective	2004–2010	249	48 y	≥6M	Kleiner et al. (2014)
12M	12.4%	Prospective	2004–2011	598	51.4 y	24M	Fontana et al. (2015)
6M	18.8%	Prospective	2004–2011	598	51.4 y	6M	Fontana et al. (2014)
6M	17.5%	Prospective	2004–2013	899	—	≥6M	Chalasani et al. (2015)
6M	13.0%	Retrospective	2012–2014	25,927	40–49 y	≥6M	Shen et al. (2019)

**Abbreviations:** HC, hepatocellular injury; Chol, cholestatic injury; Mix, mixed injury.

## 4 Clinical Manifestation

**FIGURE 1:**
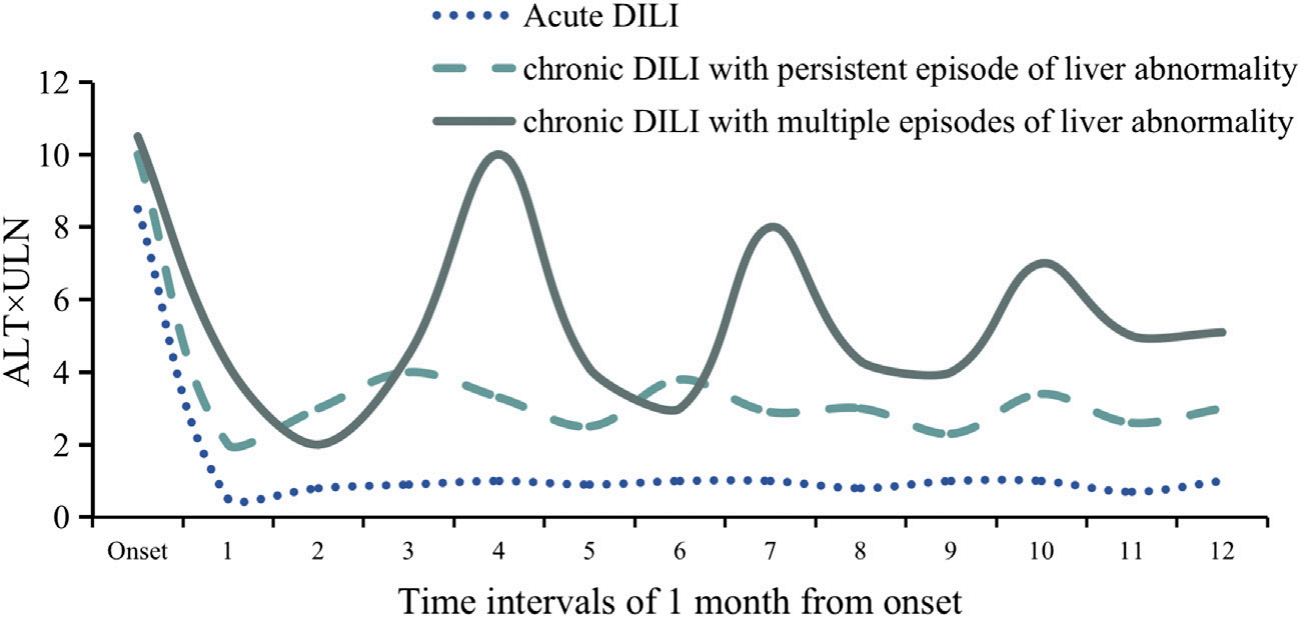
Schematic diagram of ALT fluctuation in three classic patients with acute DILI, chronic DILI with persistent episode of liver abnormality, and chronic DILI with multiple episodes of liver abnormality. ALT values during the 12 months from DILI onset. Each time interval includes 1 month.

In clinical practice, some chronic DILI patients had biochemical indicators that fluctuated up and down from the normal level and even went back to the value of the first DILI episode (European Association for the Study of the Liver, 2019) Interestingly, a part of the cases presented multiple episodes in the follow-up without conditions of drug re-challenging (European Association for the Study of the Liver, 2019) and drug recurrence (Lucena et al., 2011) (Figure 1). According to pathogenic drugs and existing pathological manifestations, ten subtypes of chronic DILI were summarized, such as autoimmune-like DILI (AL-DILI), vanishing bile duct syndrome (VBDS), drug-induced steatohepatitis (DISH), and sinusoidal obstruction syndrome (SOS) (Kleiner 2018). In clinical practice, chronic DILI can represent in many phenotypes and mimic a series of clinical liver diseases, which causes difficulty in diagnosing a specific phenotype. In this part, we will discuss five manifestations, and they may be confronted with challenges in diagnosis or need to be recognized or paid attention early.

### 4.1 Autoimmune-like Drug-Induced Liver Injury

AL-DILI can imitate AIH (Hisamochi et al., 2016), which has some challenges in disease management, diagnosis, and treatment. AL-DILI patients present incorporate high titer of autoantibodies (Bjornsson et al., 2010), such as anti-nuclear antibodies (ANA), anti-smooth muscle antibodies (SMA), and anti-mitochondrial antibodies (AMA), liver immune cell infiltration (Foureau et al., 2015) and/or IgG (Stine and Northup 2016), and/or other positive features that appear in the diagnostic criteria of AIH (Chinese Society of Hepatology and Chinese Society of Infectious 2017). However, autoantibodies are usually not disease-specific (Sebode, Schulz, and Lohse 2017), and it is not easy to distinguish between AL-DILI and AIH. As for histological results of the liver, specific cell types (lymphocytes and neutrophil) infiltrating the hepatic portal vein may offer evidence to precisely diagnose patients with AL-DILI (Foureau et al., 2015). Furthermore, the outcome of corticosteroid treatment appears to be an identification that the disease does not recur after stopping the application of corticosteroid (Foureau et al., 2015; Stine and Northup 2016; Sebode et al., 2017; Czaja 2011). However, some cases of drug-induced AIH do not show biochemical reactivation. Of note, there is still no consensus on the types of immunosuppressive regimen and the use. The current view is that after the withdrawal of corticosteroid therapy, it is necessary to closely monitor immunoglobulins such as IgG (once-a-week follow-up for the first 1–2 months, once every 2–3 weeks for the next 2–3 months, and every 3 months for the next 1–2 years) (Sebode, Schulz, and Lohse 2017). Furthermore, some participating drugs have reported in previous studies, which are also evident indications: nitrofurantoin (Bjornsson et al., 2010; Martinez-Casas et al., 2018; Licata et al., 2014), NSAIDs (Bjornsson et al., 2010; Martinez-Odriozola et al., 2010), minocycline (De Boer et al., 2016; Suzuki et al., 2011), α-methyldopa (Bjornsson et al., 2010), hydrazine (Bjornsson et al., 2010), ipilimumab and TNF-α antagonists (Stine and Northup 2016; Natalia et al., 2012) (Table 3). Besides, the minocycline or nitrofurantoin accounted for a large proportion of offending drugs about AL-DILI, and about 50% of cases caused by methyldopa and hydralazine have a phenotype of autoimmunity similar to AIH (De Boer et al., 2016).

**TABLE 3:** Drugs associated with chronic DILI sub-types.

Chronic DILI sub-types	Associated drugs
Autoimmune-like DILI (AL-DILI)	Nitrofurantoin, NSAIDs, minocycline, α-methyldopa, hydrazine, ipilimumab and TNF-α antagonists
Vanishing bile duct syndrome (VBDS)	Amoxicillin and clavulanate potassium, ibuprofen, atovaquone/guanine, pembrolizumab, infliximab, pesidatinib (PLX3397) + paclitaxel, meropenem, efavirenz, trimethoprim/sulfamethoxazole and temozolomide
Drug-induced steatohepatitis (DISH)	Antiarrhythmic drugs, methotrexate, tamoxifen, valproic acid, nucleoside reverse transcriptase inhibitors and chemotherapy
Sinusoidal obstruction syndrome (SOS)	Pyrrolizidine alkaloids (PAs), hematopoietic stem cell transplantation, oxaliplatin

Exploring the molecular mechanism of AL-DILI has become an expected breakthrough. At present, AL-DILI and AIH have similar molecular mechanisms, including gene polymorphism of human leukocyte antigen (HLA), effector T cells mediating liver inflammation, and pro-inflammatory factors in the liver (Grove and Aithal 2015; Sebode et al., 2017). The relationship between immune checkpoints (such as IL-10 signaling pathway (Aithal et al., 2004; Pachkoria et al., 2008), eosinophils (Watkins and Seeff 2006), CTLA-4 (Metushi et al., 2015), PD-1 (Metushi et al., 2015), and Cbl-b (Metushi et al., 2015)) and AL-DILI has been preliminarily confirmed, however, the single knockout of these molecular checkpoints did not lead to permanent immune deficiency.

For another aspect, many opinions hold that negative regulatory immune components involved in the formation of immune tolerance play an important role in the pathogenesis of AL-DILI, especially the role of Treg cells. For example, the number of Treg cells is inherently small, which leads to innate defects of negative immune regulation (Moises 2012), therefore, even after drug withdrawal, a small amount of inflammation will continue to appear in the liver. However, the role of Treg cells in AL-DILI is not clear and it is worth to explore further.

The current consensus on AL-DILI is that most of the active ingredients are formed after drug metabolism (Faulkner et al., 2014), so differences in pharmacokinetics and immune system functions in the population are likely to affect the occurrence of AL-DILI. Thus, it is necessary to analyze the kinetic mechanism of drugs in the human body. As AL-DILI and AIH overlap both in clinical manifestations and molecular mechanisms, more in-depth exploration and study of their mechanisms can improve the understanding of AL-DILI and help in the discovery of targeted treatments.

### 4.2 Vanishing Bile Duct Syndrome

Early diagnosis of VBDS is difficult and relies on liver biopsy. This syndrome usually has a delayed onset (mostly after 1–6 months of medication), which is related to different degrees of liver injury (Bethesda 2012). A study from DILIN reported that 26 of 363 (7%) DILI patients who underwent liver biopsy presented with different degrees of bile duct loss (Bonkovsky et al., 2017). Notably, some studies have shown that drug-induced VBDS has no significant relationship with drug dosage (Xie et al., 2018). In other words, although there may be a short-term biochemical improvement after the culprit drug is stopped, ductopenia still exists continuously. Degott et al. stated for the first time in 1992 that ductopenia might be the consequence of acute cholangitis, and emphasized that the severity of the early acute bile duct injury is closely related with the degree of ductopenia and the chronicity of the disease (Degott et al., 1992). So far, there are no effective drugs to control bile duct injury. Prevention and early detection of ductopenia are necessary and it is suggested that patients with severe cholestatic DILI should undergo imaging of the biliary tree and liver biopsy (Grewal and Ahmad 2019).

It is reported that before bile ducts disappeared, the liver might have experienced acute cholangitis which is related to immune allergy (Bethesda 2012). Bile duct cells can express a variety of Toll-like receptors (TLR) that initiate a cascade signal to recruit T cells, macrophages, and natural killer T (NKT) cells to respond to biliary infections, after being activated by pathogens (Zagory et al., 2015). Genetic HLA variants are particularly related to hypersensitivity reactions in bile duct diseases induced by specific drugs (Nicoletti et al., 2017). Bile duct cells constitutively express HLA class I molecules that are key proteins in regulating T cell-mediated immunity. bile duct cells also express HLA class II molecules in the cholestatic disease and HLA class II molecules have antigen-presenting cell (APC)-mode activity (Zagory et al., 2015). Moreover, bile duct cells can be exposed to the lipid antigens of bile, which may trigger immune responses of NKT cells (Schrumpf et al., 2015).

Besides, CD1d presentation in basolateral may be a sensor in the exposure of bile duct cells on lipid antigens. CD1d relies on its affinity with ligands to detect changes in lipid content in the cell. There is a view that new glycolipids induced by foreign antigens could activate NKT cells and these NKT cells can rapidly release pro-inflammatory cytokines to activate the immune system (Joyce 2001). This complex immune response makes bile duct cells more vulnerable to immune-mediated attacks, especially in those individuals who are sensitive to immunogenicity.

Most of the drugs that cause VBDS are related to the susceptibility of HLA, and can support the role of activated T cells in the molecular mechanism of liver injury (Table 4). Implicated classes of agents comprise antineoplastic drugs, macrolide antibiotics, penicillins, sulfonamides, fluoroquinolones, antifungals, NSAIDs, phenothiazines, tricyclics antidepressants, naproxen and aromatic anticonvulsants (Flynn and Demling 1982; Bethesda 2012; Li et al., 2019). Drugs related to VBDS reported in the past two years include amoxicillin and clavulanate potassium (Li et al., 2019), ibuprofen (Park et al., 2020), atovaquone/guanine (Abugroun et al., 2019), pembrolizumab (Doherty et al., 2017; Thorsteinsdottir et al., 2020), infliximab (Shah et al., 2019), pesidatinib (PLX3397) + paclitaxel (Piawah et al., 2019), meropenem (Zubarev et al., 2020), efavirenz (Nwaesei et al., 2019), trimethoprim/sulfamethoxazole (Kathi et al., 2020) and temozolomide (Bethesda 2012) (Table 3), which provides a clue for the early detection of VBDS in clinical practice.

**TABLE 4:** Drugs associated with bile duct injury and immunogenic susceptibility.

HLA gene type	Associated drug	References
DRB1*1,501	Amoxicillin-clavulanate	*J Clin Transl Hepatol.* 2019 Aggarwal et al. (2019)
HLA-B*5,701	Flucloxacillin	*Clin Pharmacol Ther.* 2019 Nicoletti et al. (2019)
HLA-A*3,301	Terbinafine	*Gastroenterology.* 2017 Nicoletti et al. (2017)

### 4.3 Drug-Induced Steatohepatitis

In recent decades, fatty liver disease has become a major burden around the world. Drugs, as a pathogenic factor, occupy a certain proportion (Wang et al., 2014). Data from 2005 showed that DISH is a rare form of DILI, and less than 2% of nonalcoholic steatohepatitis (NASH) cases are attributed (Flynn and Demling 1982)to drugs. Recent data from the Drug Induced Liver Injury Network (DILIN) indicated that although this is rarely described as the dominant pattern, 26% of cases showed some degree of steatosis, with macrovesicular steatosis as the dominant pattern in over 70% of the cases (Kleiner et al., 2014). A handful of compounds were confirmed that they can stimulate the development of steatohepatitis through their toxicity to hepatocyte mitochondria, inhibition of beta-oxidation, mitochondrial respiration, and/or oxidative phosphorylation (Schumacher and Guo 2015). The following drugs are identified as associated with DISH: antiarrhythmic drugs, methotrexate, tamoxifen, valproic acid, nucleoside reverse transcriptase inhibitors, and chemotherapy (Table 3) (Schumacher and Guo 2015; Dash et al., 2017) Also, amiodarone, perhexiline, Bis (2-Ethylhexyl) maleate, and diethylamino ethoxyh exestrol are known to directly cause liver steatosis (Dash et al., 2017). Some drugs were reported to be associated with fat deposition, like tamoxifen, cisplatin, and irinotecan (Schumacher and Guo 2015). Non-steroidal anti-inflammatory drugs can also affect liver fat distribution through enterohepatic circulation (Massart et al., 2017). A prospective study focused on the incidence and risk factors for non-alcoholic steatohepatitis reported that those overweight and obese women with features of metabolic syndrome are prone to develop into non-alcoholic steatohepatitis but the disease, in both the tamoxifen and the placebo group, after 10 years of follow up seems to be inactive (Bruno et al., 2005). Thus, the association between tamoxifen and non-alcoholic steatohepatitis needs more exploration.

DISH is similar to other metabolic, viral, and genetic causes of non-alcoholic fatty liver disease and steatohepatitis (NAFLD and NASH) in some aspect, which makes its differential diagnosis challenging (Patel and Sanyal 2013). Although patients with DISH have a clear history of medication, the relationship between DISH and “primary” NAFLD is particularly important, because some drugs (such as methotrexate, tamoxifen, and glucocorticoid) can worsen underlying NAFLD through their metabolic effect (Grieco et al., 2005). According to clinical experience, DISH may occur several months after taking the suspected drug, but it is difficult for patients to recover from the disease within a short period after drug withdrawal. Liver biopsy is considered for patients who are at risk of DISH, because it may offer further information in an attempt to assess a patient's liver injury, though it is not considered a routine part of the clinical evaluation. Additionally, the diagnosis of DISH requires comprehensive causality assessment to rule out other possible causes and determine its association with suspected drugs. To achieve a diagnosis, monitoring, and severity assessment of DISH, researchers have been concurrently exploring and identifying DISH biomarkers, such as lipid droplets selective probes (Cho et al., 2020).

### 4.4 Sinusoidal Obstruction Syndrome

SOS, also known as a hepatic veno-occlusive disease (HVOD), is a vascular disease of the liver, which is characterized by oedema and necrosis of endothelial cells in the hepatic sinusoids, hepatic venules, and interlobular veins to form microthrombus, which leads to congestion-induced liver injury and portal hypertension (Helmy 2006). Significant differences in etiology and diagnosis between China and western countries make SOS a special subtype. In China, SOS is mainly related to the consumption or intake of plants containing pyrrolizidine alkaloids (PAs); while in western countries, it is a potentially life-threatening complication, mainly related to hematopoietic stem cell transplantation (Table 3) (Lin et al., 2011; Vreuls et al., 2016; de Ledinghen et al., 2020). SOS is characterized by jaundice, painful hepatomegaly, and ascites, so SOS is non-specific and difficult to diagnose in the early stage, which is often confused with Budd-Chiari syndrome (Zanetto et al., 2019). The diagnosis of SOS requires comprehensive judgment combined with CT or/and MRI. However, in Europe, clinical criteria (Seattle criteria and Baltimore criteria) (McDonald et al., 1984; Jones et al., 1987; McDonald et al., 1993) have been established to aid in the diagnosis and classification of SOS, rather than histologic or hemodynamic confirmation (Mohty et al., 2015). Also, oxaliplatin has been reported worldwide as a possible cause of SOS (Vreuls et al., 2016; Vigano et al., 2017).

## 5 Risk Factors

In recent years, with the increasing incidence of chronic DILI, experts have begun to explore risk factors that can predict chronic DILI to prevent and detect it early. Several clinical trials have provided evidence that some risk factors are related to chronic DILI. Among these risk factors, we mainly discuss the following four risk factors: age, gender, the severity of the first attack, and levels of alkaline phosphatase (ALP) and total bilirubin (TB).

### 5.1 Age

For almost all chronic diseases, elderly patients are always concerned because of their deficient immunity and the declined function of metabolism (Dugan et al., 2020). This is also the case in the area of chronic DILI. Currently, there is no global report about the elderly population in chronic DILI. Existing research mainly comes from the US-DILIN network, Spain, and Iceland, respectively. Although evidence provided in these studies indicates that the elderly population can be regarded as an independent risk factor for chronic DILI, there are differences in the age of the included population (Fontana et al., 2014; Kleiner et al., 2014; Fontana et al., 2015; Medina-Caliz et al., 2016). According to Fontana’s report, the average age of the middle-aged group was 52.6 years in DLIIN registry (Fontana et al., 2015). In China, Zhu et al. reported the age of the elderly population to be more than 50 years in inpatients with DILI (Zhu et al., 2019). In Spanish DILI registry, the average age of the study population of DILI was 63 years (Medina-Caliz et al., 2016). However, the above results are insufficient to explain whether the elderly population older than 50 years can be as an independent risk factor. Therefore, large sample size studies are needed to make further comparisons among different age groups.

### 5.2 Sex

Previously, the relationship between sex-related risk and chronic DILI has not been confirmed; however, more recent studies have raised concern for it. Female DILI patients seem to be more likely to develop chronic DILI from the acute DILI episode (Fontana et al., 2014; Medina-Caliz et al., 2016). Some scholars postulated that some kinds of special periodic changes of estrogen and progesterone levels in the female population may have an impact on their immune function (Moulton 2018). There is a study reporting that sex and a proxy of menopause were related to various features of inflammation and injury in DILI (Suzuki et al., 2017). Therefore, females DILI patients who are in or about to enter menopause may have some risks suffering from chronic DILI (George et al., 2018). Moreover, in our study about glucocorticoids treatment for chronic DILI patients, we observed that there was much more elder female in the cohort met the inclusion criteria. Sex and sex hormones can regulate drug metabolism and transport and therefore influence the host response to xenobiotics (Waxman and Holloway 2009; Yang et al., 2012). Sexual dimorphism in cell stress, cell autophagy, cell death, immune response, inflammation and tissue repair has been proved in many systems (Beagley and Gockel 2003).

### 5.3 Severity of the First Attack and Levels of Alkaline Phosphatase and Total Bilirubin

A more serious initial attack in DILI was an independent risk factor for chronicity (Medina-Caliz et al., 2016). Meanwhile, a more serious onset of DILI may also show the high level of ALP and TB. According to the report, at the second month after the onset of DILI, the levels of ALP and TB (ALP exceeded 1.1 times of the upper limit of normal value (ULN) and TB exceeded 2.8ULN) had reference value in predicting chronicity (Medina-Caliz et al., 2016). Moreover, two studies from the same team indicated that the ALP level both at the first onset of DILI and at the 6th month after the onset have a predictive value of chronic DILI (Fontana et al., 2014; Fontana et al., 2015). A report from China also found a certain association between TB and chronic DILI. The result showed that T_0.5TBIL_ (the time interval that the value of TB falls to the half of its peak) is an early independent predictor of chronic DILI (Zhu et al., 2020). Therefore, the severity of the first attack and levels of ALP and TB may reflect the chronic trend at an early stage.

### 6 THE ROLE OF LIVER BIOPSY IN CHRONIC DRUG-INDUCED LIVER INJURY

Chronicity of DILI is an important issue in clinical practice. The persist abnormality of liver biochemistries over one year from the onset is as one of the characteristics to detect and define chronic DILI, however, the diagnosis is still challenging. Some DILI cases may be accompanied by preexisting liver diseases, for example, chronic alcoholic liver disease. According to studies, 4.1%–10% of DILI cases with preexisting liver diseases in all included patients (Andrade et al., 2005; Bjornsson et al., 2013; Chalasani et al., 2015). Thus, it is necessary to distinguish chronic DILI from other preexisting chronic liver diseases. Even if a liver biopsy is not required for the diagnosis of chronic DILI, it may bring some useful information about the selected DILI patients and offer diagnosis information when some DILI cases fail to resolve several months after stopping the offending drugs. A liver biopsy may be taken into account in the following conditions: 1) a case was suspected as chronic DILI but the causal relationship is weak, 2) a case was suspected as chronic DILI but it may be accompanied with a chronic underlying liver disease that is possibly reactivated, 3) a DILI patient suffers from (multiple DILI episodes without drug-rechallenging, 4) the imaging evidence of DILI patients showed early signs of liver cirrhosis.

### 7 THE APPLICATION OF GLUCOCORTICOIDS IN CHRONIC DRUG-INDUCED LIVER INJURY

After drug cessation, some DILI patients may progress into chronic DILI even cirrhosis. In the Spanish DILI Registry study, 8% of DILI patients suffered from sustained liver injury longer than one year, among which 64% could not resolve after three years. Subsequently, 44% of those long-term unsolved DILI patients developed into cirrhosis(Medina-Caliz et al., 2016). Therefore, effective drugs that can be used to halt the progression of chronicity are necessary. Since the immune mechanism has a significant influence in the pathogenesis of patients with chronic DILI, glucocorticoids have been considered in some patients with marked signs of autoimmunity. Glucocorticoids are stated as the treatment to help manage DILI patients with hypersensitivity or autoimmune-like symptoms in China DILI Guidelines (2017 version) (Yu et al., 2017) and the European DILI Guidelines (2019 version) (European Association for the Study of the Liver, 2019). However, the application of glucocorticoids in chronic DILI lacks high-level medical evidence. In our RCT study (NCT02651350) about glucocorticoids treatment for chronic DILI patients, we observed that chronic DILI patients with multiple episodes responded well to glucocorticoid (unpublishing data). Glucocorticoids have anti-inflammatory, detoxification, anti-allergic, anti-shock, nonspecific immunosuppression, and antipyretic effects, which may help to alleviate inflammation and pathological immune reactions (Wang et al., 2020; Kan and Himes 2021). In the pathogenesis of chronic DILI, disorders of immuno-regulation have been taken into consideration especially in AL-DILI and chronic DILI with multiple episodes (Mak and Uetrecht 2015). Based on our clinical experience, glucocorticoids may be considered in the treatment for chronic DILI patients with ALT>10×ULN, or ALT>5×ULN and TBIL>2×ULN. However, the reports of chronic DILI cases successfully treated with glucocorticoids are less and the application of glucocorticoids in other special forms of chronic DILI has not been confirmed. To verify the effect of glucocorticoids in chronic DILI, a large-size RCT conducted by multiple centers is needed.

## 8 Conclusion

Chronic DILI is a kind of complex chronic liver disease which lacks comprehensive research and remains challenging in clinical practice. Over the past 30 years, studies conducted by the US-DILI Network, Spain, Iceland, and other international institutes have expanded our knowledge of chronic DILI. Available guidelines on definitions of chronicity and defined chronic clinical phenotypes can assist the diagnose of chronic DILI. In DILI patients, especially those with multiple episodes, it is meaningful to consider the conversion from acute DILI to chronic DILI. The variants of chronic DILI should be also highly suspected in those patients who present specific manifestations. Certain drugs or herbals, such as valproic acid, pembrolizumab, antiarrhythmic drugs and the herb of chrysanthemum-like groundsel (Pas) have hepatotoxicity and they may be involved in some forms of chronic DILI. A thorough investigation of the history of those medications may help the diagnosis of chronic DILI. Risk factors for predicting chronic DILI are important and they may be helpful for early detection of chronicity. Future research in personalized medicine will help identify and mitigate the risk of chronic DILI after the DILI onset.
