# Supplementary material for: Chronic Drug-Induced Liver Injury: Updates and Future Challenges
Source: Front Pharmacol. 2021 Mar 8;12:627133. doi: 10.3389/fphar.2021.627133 (PMC7982586; doi:10.3389/fphar.2021.627133)
Supplement: Supplementary file 1 [file datasheet1.pdf]

Figure 1 Schematic diagram of biochemical index changes in patients with chronic DILI

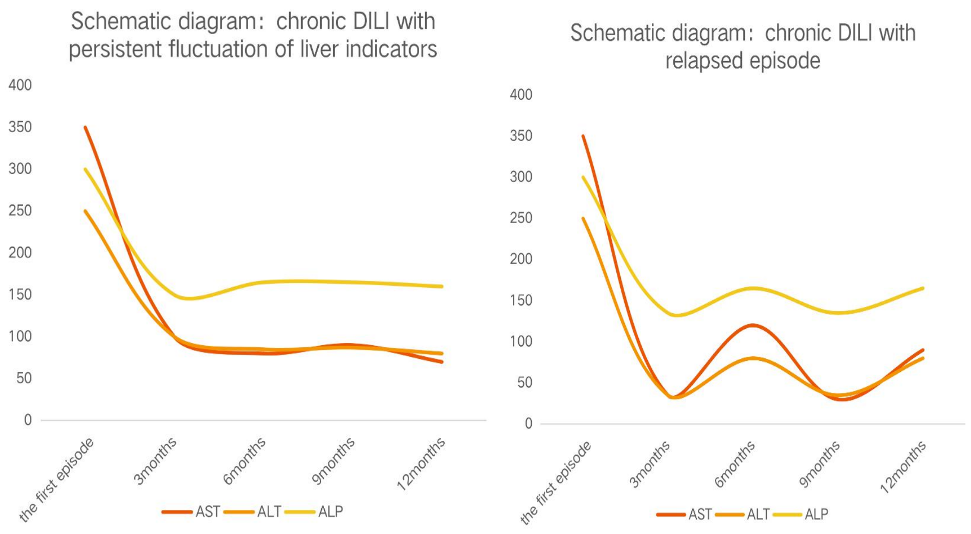

Abbreviations: AST: aspartate transaminase; ALT: alanine aminotransferase; ALP: alkaline phosphatase
